# On the Road to Accurate Biomarkers for Cardiometabolic Diseases by Integrating Precision and Gender Medicine Approaches

**DOI:** 10.3390/ijms20236015

**Published:** 2019-11-29

**Authors:** Letizia Scola, Rosa Maria Giarratana, Salvatore Torre, Vincenzo Argano, Domenico Lio, Carmela Rita Balistreri

**Affiliations:** 1 Department of Biomedicine, Neuroscience and Advanced Diagnostics (Bi.N.D.), University of Palermo, 90134 Palermo, Italy; letizia.scola@unipa.it (L.S.); rosamaria.giarratana@unipa.it (R.M.G.); domenico.lio@unipa.it (D.L.); 2 Unit of Cardiac Surgery, University of Palermo, 90127 Palermo, Italy; salvotower86@gmail.com (S.T.); vargano@hotmail.com (V.A.)

**Keywords:** cardiometabolic diseases, management, multi-omics integration, gender medicine, accurate CMD biomarkers

## Abstract

The need to facilitate the complex management of cardiometabolic diseases (CMD) has led to the detection of many biomarkers, however, there are no clear explanations of their role in the prevention, diagnosis or prognosis of these diseases. Molecules associated with disease pathways represent valid disease surrogates and well-fitted CMD biomarkers. To address this challenge, data from multi-omics types (genomics, epigenomics, transcriptomics, proteomics, metabolomics, microbiomics, and nutrigenomics), from human and animal models, have become available. However, individual omics types only provide data on a small part of molecules involved in the complex CMD mechanisms, whereas, here, we propose that their integration leads to multidimensional data. Such data provide a better understanding of molecules related to CMD mechanisms and, consequently, increase the possibility of identifying well-fitted biomarkers. In addition, the application of gender medicine also helps to identify accurate biomarkers according to gender, facilitating a differential CMD management. Accordingly, the impact of gender differences in CMD pathophysiology has been widely demonstrated, where gender is referred to the complex interrelation and integration of sex (as a biological and functional marker of the human body) and psychological and cultural behavior (due to ethnical, social, and religious background). In this review, all these aspects are described and discussed, as well as potential limitations and future directions in this incipient field.

## 1. Challenges and Drawbacks in the Research of Appropriate Biomarkers for the Management of Cardiometabolic Diseases

Cardiometabolic diseases (CMD) represent a heterogenous group of diseases, characterized by difficult management, as well as limited prediction, based essentially on classical risk factors because accurate molecular predictors are lacking [1,2]. Therefore, the search for appropriate biomarkers with diverse applications from risk prediction and screening to diagnosis and prognosis, and the creation of specific algorithms useful in preclinical and clinical settings are encouraged. To date, different biomarkers have emerged in the literature. For example, the interesting data obtained from different studies on the role of some circulating molecules, such as C-reactive protein (CRP) [3,4,5], adiponectin [6,7,8], leptin [9,10,11,12,13], in quantifying the CMD risk, have failed to identify promising biomarkers for CMD prevention. Indeed, they have not been confirmed in large meta-analyses or by using an association’s analyses (i.e., Mendelian randomization analyses) [3,4,5,6,7,8,9,10,11,12,13]. Nevertheless, novel biomarkers continue to be suggested by CMD research groups which include: inflammasome molecules [14,15], cardiac fibrosis markers [16], apelin [17], cytokines [2], metabolites [18], and microRNAs [19,20,21], etc. However, their biological role in CMD reduction, diagnosis, or prognosis remains to be elucidated. Such research appears difficult, because several steps in the investigations are required, which include: (a) assessing associations with preclinical and clinical phases of CMD, (b) confirming their replication in numerous studies, and (c) testing their effective clinal utility before affirming any definitive statement on their potential relevance in clinal CMD management. In addition, combinations of biomarkers and panels of biomarkers, rather than a single biomarker, are required to improve CMD prediction, diagnosis, and prognosis by creating algorithms. Studies on the therapeutic effects of potential biomarkers could also be promising. However, such investigations generally show numerous limitations, essentially linked to high biological (i.e., sex, age, and genetic background, ethnicity, epigenetics, microbiome and environmental factors) and methodological variability. Moreover, biomarker appraisal in large population study results are more difficult, because the biomarkers identified may be the result of diverse pathophysiological processes, and therefore it is crucial to evaluate the reliability, validity, sensitivity, specificity, ascertainment bias, and the analysis of data. Consequently, any potential confounding factors need to be revealed that in the are not easy to recognize in the major part of such studies. From recent studies, the emerging biomarkers that are not well investigated in the population’s research are of interest. Indeed, they are affected by the multiethnic origins of the populations studied, the diverse pathophysiological conditions of individuals enrolled, sex and gender discrepancy, and diverse experimental conditions in the human and animal models used. Additionally, Mendelian randomization analysis on polymorphisms in biomarker genes is suggested for assessing the link of the potential associations with CMD risk and biological relationships. The omics biomarkers, whose relevance remains to be determined, are another important aspect recently suggested by the precision medicine studies. Omics biomarkers have been derived from studies based on unstandardized protocols for sample collection, storage and use. Thus, all such mentioned studies have not used standardized methodologies and, until now, no clinical trials have been planned to evaluate the effectiveness of the large list of biomarkers discovered [22,23].

**Figure 1:**
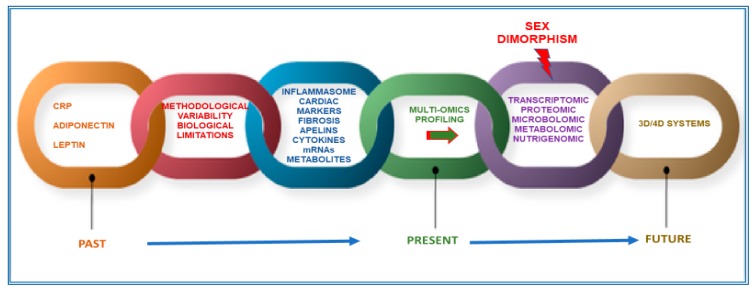
Perspective and limits for new markers of cardiometabolic diseases CMD.

All these observations suggest that further scientific efforts are needed for the identification of appropriate biomarkers for CMD. To this aim, further advances may be achieved by studying, through a new technological appraisal based on innovative approaches and systems, molecules associated with disease pathways that can represent valid disease surrogates. In order to achieve this goal, in this review, we emphasize the necessity to integrate multi-omics analyses, by combining up-to-date common investigations on genomics, epigenomics, transcriptomics, and proteomic profiles, with the recent metabolomics, microbiomics, and nutrigenomics investigations. Accordingly, some biomarkers identified as a result of these investigations are reported as examples. We also underline the importance of including, in these investigations, high-throughput technologies and innovative three-dimensional (3D) and four-dimensional (4D) systems for a deep knowledge of the molecular framework of CMD and, consequently, for identifying well-fitted biomarkers. Particular attention is also given to the discussion of the relevance of sex dimorphism, as a major challenge in the identification of accurate biomarkers, as well as in the CMD differential management and therapies. In addition, other confounding factors are considered, such as age and medication (Figure 1). Finally, we describe some gaps in these approaches, as well as potential limitations and future directions in this emerging field.

## 2. Multi-Omics Approaches, 3D and 4D Systems, and Innovative Techniques to Search for More Appropriate CMD Biomarkers: Reasons, Gaps, and Limitations

The importance of genetics to the onset of CMD is well recognized [14] but environmental factors [24] play a crucial role in defining the degree of genetic susceptibility to CMD that occurs. Continuous environmental exposures modulate the CMD risk [24], and diet is one of the most crucial [24,25]. Smoking represents another important modifiable risk factor, but advances in the conspicuous decrease of smoking rates have been achieved in several Western populations [24,26]. For example, in the United States the decrease has been significant and, consequently, there has been a reduction in CMD cases since the 1960s [26]. However, another relevant risk factor is that obesity related to diet has increased after a decrease in smoking percentages [24]. Thus, diet is both a crucial and directly modifiable risk factor [24,25]. This indicates that a better understanding of healthy nutrition can augment quality of life and decrease worldwide disease morbidity and mortality [27,28]. For example, diverse studies and recent meta-analyses have reported the crucial role of a Mediterranean diet in reducing the risk of chronic diseases, such as CMD, and improving life quality [28,29,30,31,32,33]. In addition, the key role of microbiome in CMD onset has also been demonstrated, as well as the interaction between diet and gut microbiota and CMD [34,35]. An altered diet, consequently, modifies the composition of gut microbiota, referred to as dysbiosis, which is significantly associated with CMD, including atherosclerosis, hypertension, heart failure, chronic kidney disease, obesity, and type 2 diabetes mellitus. In addition to these changes, altered metabolism of gut microbiota has also been shown to contribute to CMD development [28,34,35].

**Figure 2:**
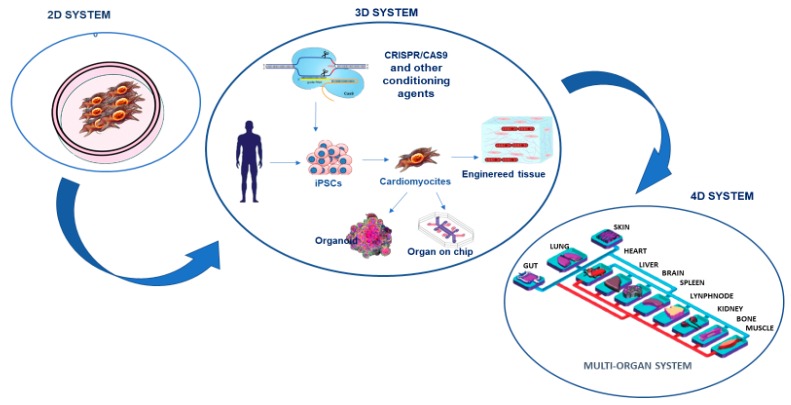
The study of cardiometabolic pathologies and the search for relative biomarkers have constantly evolved. After the two-dimensional systems constituted by monolayer cells, the so-called three-dimensional systems that combine the use of cardiomyocytes derived from the patient’s committed stem cells with artificial platforms and biosensors have been developed. The next goal is the standardization of 4D microchip systems that mimic the complexity of multi-organ interactions.

This recent evidence has motivated CMD researchers to perform not only genetic studies, but also other omics investigations, including the analysis of the transcriptome, epigenome, proteome, and more recently, the evaluation of metabolome, microbiome, nutrigenomics, etc. However, the current omics studies on CMD have focused on individual omics types that have provided data on a small part of the molecules involved in the complex CMD mechanisms, whereas the solution could be derived from their integration that could lead to obtaining multidimensional data. Such data could provide a better understanding of the molecules related to CMD mechanisms and, consequently, increase the possibility of identifying well-fitted biomarkers [36]. This could also facilitate their complex management and therapy, the most important objectives of clinical community. Certainly, the use of more appropriate study models, such as the innovative three-dimensional (3D) cardiometabolic tissue constructs, can help in the integration of multi-omics approaches [37,38] (see Figure 2). The 3D systems attempt to address limitations of the conventional two-dimensional (2D) in vitro models, such as monoculture cellular assays, as well as in vivo animal models [37,38,39]. The 2D in vitro models have limitations in the simulation of CMD pathophysiology because of the high degree of complexity in the structure and function of the cardiometabolic tissues [37,38]. Precisely, these analyses are unable to summarize the complex cell-extracellular matrix (ECM), cell–cell, and tissue-level interactions. To address the limitations of 2D assays, animal (e.g., mouse) models have been used, because they mimic the complicated tissue-level representation. But such investigations have led to the awareness of numerous differences between mouse models and human disease [40,41]. Currently, several techniques for creating 3D cardiometabolic tissue models have been proposed. In addition, recently, in vitro research has also suggested the use of human-derived cardiac-metabolic cells in 3D systems, derived from methods for cardiometabolic differentiation of human stem cells in order to generate patient-specific and genetically edited cardiometabolic cells [42]. Specifically, recently, human induced pluripotent stem cells (iPSCs) have also been used as an attractive approach for overcoming the above-mentioned limitations of animal models, creating a more appropriate CMD model and enabling discovery drugs [43]. This is strongly linked to their features. The iPSCs not only have the capacity for self-renewal and differentiation but can also be directly produced from the patients’ skin fibroblasts, blood cells, and other somatic cell sources. Consequently, patient-specific iPSCs could provide a source of unlimited disease-relevant cells in a personalized manner, including cardiomyocytes, which have been previously inaccessible. However, to use iPSCs, their genome instability, epigenetic memory associated with the reprogramming process [28,44], and their maintenance represent important limitations to their effective application. Indeed, these factors exemplify the very issues in achieving the integrity of iPSC cellular derivatives and creating accurate models of diseases [43]. In addition, iPSCs are commonly used in 2D monolayers to discover disease phenotypes, however, the 2D systems do not reproduce the tissue- and organ-level structures and functions. This has led to the development of 3D iPSC models that can more accurately reproduce tissue- and organ-level disease pathophysiology (see Figure 2). Furthermore, innovative biomaterials, as well as micro- and nanoscale technologies can be used as components of 3D systems that enhance the functionality of engineered cardiometabolic tissues through an accurate control on cell–cell and cell-ECM interactions [37,38]. The 3D micro-engineered cardiometabolic tissue models have been successfully utilized to better understand the biological basis of CMD progression and to increase the effectiveness of pharmaceutical testing of candidate therapeutics to help prevent or reverse CMD [37,38]. The above-mentioned 3D systems show some limitations for studying both ontogenesis and disease progression because they are intrinsically dynamic processes with cellular and tissue-level biological activities altered not only spatially, but also temporally [45]. Currently, the 3D existing systems principally reproduce spatial events in a single organ and fail to show associated temporal events, including the progression of organ development, ageing, the dynamics of tissue healing and regeneration, the exchange of metabolites between organs, and the sequential pathogenesis of inflammation, infection and multi-organ failure [45]. Thus, further advances in the reproduction of temporal events in the existing 3D models are encouraged. As an alternative technology, organ-on-chip technology can include various patient-specific iPSC-derived 3D constructs in a dynamic system, creating a 4D multi-organ system, also known as “body-on-chip”, via a circulating flow that mimics the systemic interactions among different tissues and organs (Figure 2). The 4D multi-organ systems can also have in-line sensors and fluorescent reporters for the real-time quantification and analysis of cellular dynamics during a disease study. The 4D multi-organ systems are, however, in the early developmental phases. Currently, they have been used to study the adsorption, distribution, metabolism, elimination, and toxicity of drugs [46,47,48]. Recently, a proof-of-principle system comprising intestine, skin, liver, and kidney chips has been developed, although it was only maintained for more than four weeks [49]. In the future, 4D multi-organ systems are expected to be further improved by integrating with different 3D platforms to create efficiently engineered organoid-on-chips. These could be maintained in a physiological environment produced by integrated engineered organoids for extended periods of time to enable the temporal investigation of dynamic disease pathogenesis.

Other technologies have been added to 3D systems to improve their effectiveness, such as gene-editing techniques. Among the most recent, clustered regularly interspaced short palindromic repeats-associated protein 9 (CRISPR/Cas9) and chemically modified RNA technique, have also been incorporated in 3D models in order to study related pathologies or potential disease correction through restoration [50]. For example, CRISPR-Cas9 technology allows single nucleotides to be edited with high precision in the endogenous genome, opening up the possibility of producing mouse models harboring identical gene variants to those identified by genome-wide association studies (GWAS), in humans. This is shown by studies on obesity-associated genes, where the rs1421085 T-to-C single nucleotide polymorphism has been found to favor the switch of BAT to WAT adipose tissue, reducing mitochondrial thermogenesis and increasing lipid storage. The CRISPR-Cas9 replication of the T-to-C in the equivalent primary adipocytes from a non-risk allele carrier has simulated this switch [51]. Other studies have also combined iPSCs and CRISPR/Cas9 technology to investigate the molecular and cellular mechanisms underlying inherited CMD [52]. Through these technologies, therefore, there is a belief that CMD genetical modeling could prosper. In addition, CMD researchers are also planning to include CRISPR/Cas9 assay in the final step of a deep investigation that, first, includes GWAS, which is able to identify novel genetic loci associated with CMD risk in diverse populations, and omics profiling (transcriptomics, proteomics, metabolomics, and microbiomics) studies, to translate genetic and molecular findings for clinical applications through the identification of appropriate biomarker panels, and for treatment of both rare and common CMD [53] (see Figure 2).

### 2.1. A Focus on Metabolomics and Metabolite Profiling and Examples of Related CMD Biomarkers

The tradition current omics analyses (i.e., transcriptomics, epigenomics and proteomics investigations), as well as the emerging metabolomics analyses for predictions are needed to obtain multidimensional results on CMD. Metabolomics represents the profiling of small molecule metabolites in biofluids, cells and tissues, or whole organisms [18]. During the past two decades, it has been the focus of a rapid technological evolution (i.e., magnetic resonance and various types of mass spectrometry) [18]. Such innovation has led to the application of metabolomics to define predictive biomarkers for CMD, and increasingly, as a blueprint for understanding their pathophysiologic mechanisms [18,54]. Thus, some studies have tested the associations of amino acids, fatty acids, acylcarnitines, and other metabolites with CMD. For example, targeted metabolomics has discovered a signature of dysregulated metabolism of branched-chain amino acids (BCAA) in patients with different CMD [40]. The BCAAs, including leucine (Leu), isoleucine (Ile), and valine (Val) have several functions which include: (a) they are involved in synthesis of nitrogenous compounds; (b) they are also signaling molecules able to regulate glucose and lipid metabolism, as well as protein synthesis; and (c) they also contribute to the regulation of intestinal health and gut immunity interacting with a signaling network, precisely represented by phosphoinositide 3-kinase/protein kinase B/mammalian target of rapamycin (PI3K/AKT/mTOR) signal pathway [55].

**Figure 3:**
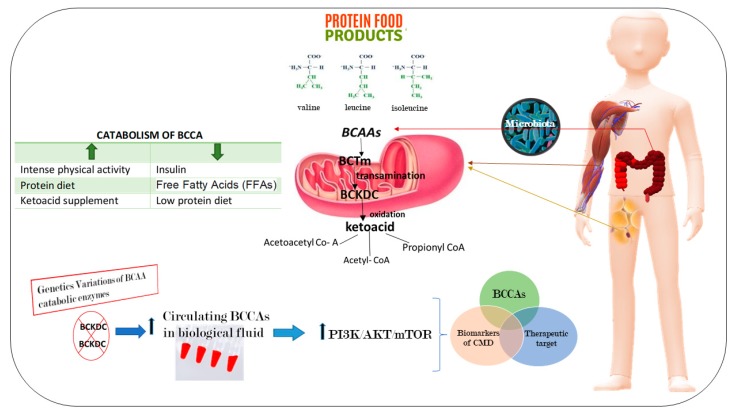
Branched-chain amino acids (BCAAs) as a novel diagnostic and prognostic metabolites of cardiometabolic diseases (CMD). The BCAAs are essential amino acids that are not synthesized in our body. Thus, they need to be obtained from food or produced by gut microbiota. At the level of peripheral tissues, mainly in muscle and adipose tissue, in the mitochondria BCAAs are catabolized. The first step is a transamination reaction performed by the mitochondrial isoform of branched-chain amino acid transaminase (BCTm). Then, they are oxidized by the multienzyme mitochondrial branched-chain-ketoacid dehydrogenase complex (BCKDC), that transforms the BCCAs into its relative ketoacids, energy substrates for the synthesis of sugars, proteins, and lipids. An alteration into the catabolic pathway determines an accumulation of BCCAs in the circulation, inducing the activation of the network of phosphoinositide 3-kinase/protein kinase B/mammalian target of rapamycin (PI3K/AKT/mTOR) signal pathways.

The established data on the significant relationship of BCAAs and their derivatives with CMD suggest that they are potential biomarkers. Furthermore, based on this link we infer that adjusting dietary BCAA levels could have beneficial effects on the parameters related to the reduction of CMD risk (Figure 3).

Moreover, recent evidence has also demonstrated that small metabolites are associated with a negative prognosis of CMD. This is the case for the metabolites of the nitric oxide (NO) pathway that show alterations in CMD patients complicated with chronic wounds of the lower extremities [56]. However, the full potential of metabolomics for identifying appropriate biomarkers remains to be realized. It is hoped that this interest will encourage more investigations focused on metabolomics in CMD research, and more specifically, on disease-associated metabolomic signatures for discovering novel CMD biomarkers and targets.

Other important data have been provided from lipidomics studies [57], since CMD exhibit changes in lipid biology. Indeed, lipid imbalance is closely associated with CMD, such as atherosclerosis, obesity, diabetes, and Alzheimer’s disease. Lipidomics or lipid profiling is a lipid-targeted metabolomics approach based on a complete analysis of lipids in biological systems [58]. Several distinct metabolic pathways potentially involved in lipoprotein metabolism in both healthy and disease states (obesity, insulin resistance, and fatty liver disease), as well as under lifestyle and dietary modification (fish consumption, carbohydrates, and probiotics) and lipid-modifying treatments (statins and low-density lipoprotein apheresis) have been recently discovered. In addition, a detailed characterization of lipid classes and molecular species present in plasma, as well as in lipoprotein fractions, has been recently obtained [57,58]. This suggests that lipidomics (for example, plasma lipoprotein) analysis together with other omics investigations, such as metabolomics, have enabled obtaining molecular specifics of the lipoprotein composition, which can be translated into an integrated knowledge of the structure, metabolism, and function of lipoproteins in CMD, and consequently, into appropriate biomarkers.

### 2.2. Microbiomic Profiling and Related Promising CMB Predictive Biomarker

**Figure 4:**
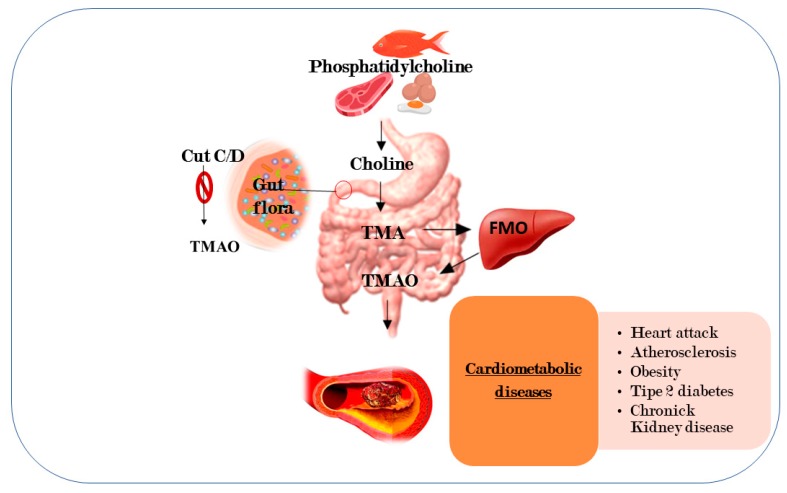
High concentration of trimethylamine N-oxide are predictors of cardiometabolic diseases. TMAO is a metabolite derived from a diet rich in fats which is metabolized to produce trimethylamine (TMA) by microbiota. The figure shows a possible mechanism for the inhibition of TMA formation by blocking the pair of microbial enzymes, Cut C/D, responsible for the transformation of choline into TMA. Trimethylamine, TMA; flavin monooxygenase, FMO; and trimethylamine n-oxide, TMAO.

Gut microbiota has been recently associated with CMD through a direct action on gut immunity or altering blood levels of bioactive metabolites [59]. Accordingly, data obtained by two research groups [60,61] have demonstrated that circulating levels of trimethylamine N-oxide (TMAO), a metabolite of the gut microbiota, are CMD predictors. TMAO is a small organic compound, predominantly obtained from choline (found in foods such as red meat, fish, poultry, and eggs), metabolized by microbiota to trimethylamine (TMA), and, then, to TMAO for action of the hepatic enzyme, flavin monooxygenase 3 [62,63]. Further studies have investigated this association obtaining contrasting data. However, recent meta-analyses have reported that elevated circulating levels of TMAO and its precursors are significantly associated with increased risk of CMD, independently of traditional risk factors [62,63] (Figure 4).

Nevertheless, further studies are necessary to confirm these promising data and the role of TMAO, even if it has been reported that it mediates modifications in cholesterol (reductions in reverse cholesterol transport) and sterol metabolism, as well as in bile acid pool size and composition, and increases platelet hyperreactivity and thrombosis risk [64].

### 2.3. Nutrigenomics and Nutrigenetics Approaches in the Research of CMD Biomarkers

Given the relevance of diet in modulating the CMD risk, the application of nutrigenomics and nutrigenetics approaches are fundamental, first, to understand the interaction between the metabolic processes and the environment’s effects in CMD processes, and, secondly, to identify molecules that have key implications in CMD prevention and treatment. Accordingly, nutrigenomics and nutrigenetics, like pharmacogenomics, can detect genetic predictors of CMD-relevant responses to diet, to be applied both as potential preventive biomarkers and targets for therapeutic interventions [24]. This could also favor the inclusion of nutrigenomics and nutrigenetics in the context of personalized nutrition. However, nutrigenomics and nutrigenetics have also been the focus of intricate investigations and false promises, which, until now, have failed to demonstrate their potential. Despite this, the American Heart Association is strongly affirming the significant relationship existing among nutrigenomics and nutrigenetics, microbiome, and gene–environment interactions and their influence on CMD onset and progression [24]. Furthermore, diverse trials have been performed to investigate the potential of dietary pattern to stop hypertension and prevent CMD such as diabetes and cardiovascular diseases [24]. Among these, the PREDIMED trial, “Primary prevention of cardiovascular disease with Mediterranean diets” demonstrated that the Mediterranean diet is helpful, because it reduces the incidence of numerous major chronic diseases in individuals at high cardiovascular risk, and it mainly includes the consumption of extra virgin olive oil [65]. These promising data have led to research genes and polymorphisms involved in the gene–nutrient interaction through epigenetic factors, and interesting data have been obtained. However, the discoveries of nutrigenomics and nutrigenetics cannot be rigorously applied to CMD prevention and treatment, at this time, and the crucial results currently obtained, or in via of being produced, can only be used as a basis for enhancing the reliability of imminent research.

### 2.4. The Difficult Challenge of Integration and Interpretation of Multidimensional Data from Multi-Omics Analyses

Data obtained from the combination of multi-omics analyses can be integrated to obtain complete information from several molecular areas that could be translated into a better understanding of underlying mechanisms involved in CMD pathogenesis and the selection of biomarkers with diagnostic or prognostic values. [66]. This first implies a phase of data preprocessing to evaluate quality control and data normalization. Correct preprocessing is crucial to eliminate outliers and nonbiological variation within a data type and increase the biological comparability between data types. Currently, multidimensional data integration tools have been developed and could be selected from the following five distinct categories: clustering and dimensionality reduction-based methodologies, predictive modeling approaches, pairwise integration, network-based methodologies, and composite approaches. They are mainly created for specific combinations of data types. Their selection requires consideration of data-driven statistical patterns and biological interpretability. However, depending on the specific applications, the significance of these two aspects may vary. Therefore, before choosing an appropriate method, it is imperative to, first, understand the biological question that is being addressed, i.e., biomarker discovery or mechanistic insight. For the discovery of diagnostic and prognostic biomarkers, data pattern is the key factor, whereas biological interpretation can be less important.

Nevertheless, this field is still in its early stages, and the flexibility, effectiveness, and robustness of data integration to obtain biological insights are still limited. The limitations are principally due to the intrinsic complexity within individual datasets and between datasets, as well as technical difficulties in integrative modeling that accurately captures true biological complexity. Furthermore, the performance of the various methodologies has not been exhaustively compared, and there is a lack of general guidance in the field on best practices. To address these challenges, future efforts should focus on intimate collaborations among computational biologists, systems biologists, and experimental biologists in the following areas described below.

## 3. Adding Another Important Layer: Sex Dimorphism in CMD and its Relevance in the Research of Accurate Sex or Gender Biomarkers

**Figure 5:**
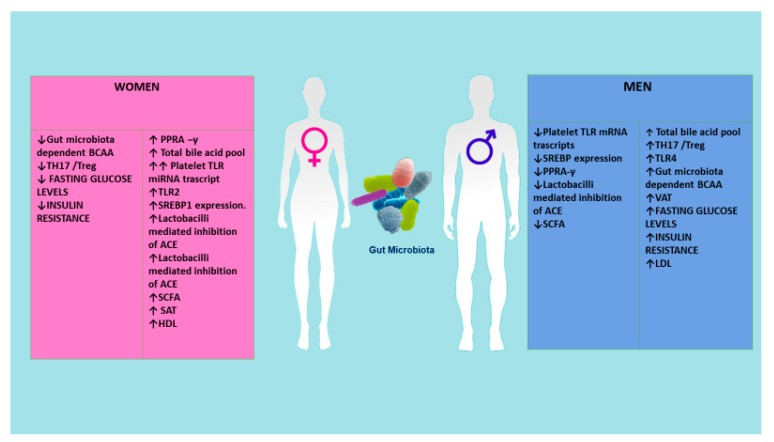
Sex differences are mediated through reversible sex hormones. Various metabolites of the microbiome can have different influences in the two genders. ACE, angiotensin converting enzyme; BCAA, branched-chain amino acid; PPRA-γ, peroxisome proliferator activator receptor-γ; SAT, subcutaneous adipose tissue; SCFA, short-chain fatty acid; SREBEP1, sterol regulatory-binding protein TLR; TLR, toll-like receptors; VCA, visceral adipose tissue.

Another important challenge in cardiometabolic research is sex dimorphism. Women and men differ not only in their anatomy and physiology, but also in more complex traits. We have recently observed these differences in lifespan (in Italy, 78.8 years for men and 84.1 years for women, respectively) [67], mortality [68], age-related diseases pathophysiology, onset, and progression [69], and immune and inflammatory responses and age-related immune and inflammatory disorders [68,69]. Recently, the sex-specific predominance of common types of CMD has also been observed. Precisely, women (and particularly women affected by diabetes) as compared with men have higher risk for heart failure, atrial fibrillation, and ischemic heart disease, even if the identification of these differences and the underlying mechanisms is only just emerging. Some studies suggest that sex hormones, sex-specific molecular mechanisms, and gender influence glucose and lipid metabolisms, as well as cardiac energy metabolism, and function [70]. Accordingly, Lusis et al. [71] have largely stressed sex differences in metabolic and cardiovascular traits, evidencing differences in body fat distribution, glucose homeostasis, insulin signaling, ectopic fat accumulation, and lipid metabolism during normal growth and in response to hormonal or nutritional imbalance. They have also affirmed that these differences can partly be mediated through sex hormones and the sex chromosome complement [71]. In addition, an intricate interaction between environmental, social structural, behavioral (i.e., the complex pattern of roles and values that define what is thought as “masculine” and “feminine”) and genetic factors have been also proposed as probable explanation [70]. Our group have underlined that diverse genetic, epigenetic, hormonal factors, and different cellular and molecular signaling pathways are implicated in the diverse sex-specific effects in women related to onset and progression of CMD [72] (see Figure 5). The group of Razavi has recently reported that whereas modest differences characterize the gut microbiome composition of women as compared with men, there appears to be strong effects of gut microbiome-dependent metabolites in females as compared with males [73] (see Figure 5). Accordingly, microbiome-associated TLR signaling pathways, bile acid metabolism, and steroid hormone modulation have been demonstrated to be the crucial drivers in sex differences in CMD risk. However, additional studies are imperative to explain the role of these pathways and to define their role as biomarkers and therapeutic targets (see Figure 5).

The evidence reported above infers suggesting the requirement of applying in women, as compared to men, diverse biomarker’s panels for CMD diagnosis, prevention, and prognosis. This finding is supported in the evidence of emerging literature. Schiebinger and coworkers. have elegantly emphasized these differences and suggested that sex and gender analysis need to be incorporated into the experimental design. This can enable advancements, such as improved treatment and differential management of CMD and insights into the societal impact of algorithmic bias [74].

## 4. Age and Medication: Other Factors to Consider in the Search of CMD Biomarkers and Strategies for Reducing their Effects

In studying diseases, such as CMD, we are often forced, by timing, economics, or ethics, to use not perfect experimental designs. This determines various interpretational problems, including the issue of confounding [75]. Confounding defines a situation in which the result is biased because of the differences between compared groups, such as baseline characteristics, prognostic factors, or concomitant interventions. For a factor to be a confounder, it must differ between the comparison groups and predict the outcome of interest. Confounding is, thereby, fundamental to the design, analysis, and interpretation of studies intended to estimate causal effects. This implies performing an appropriate selection of the populations in an investigation [75]. Precisely, this leads to considering no explicit differences (imbalances) between the populations included in a study with respect to circumstances or covariates that could affect the association of cause and effect. Covariates represent the confounders in a study. In the context of CMD studies, age and medication are valid confounders. For example, Wike and coworkers demonstrated that clinical and echocardiographic manifestation of aorta stenosis are age dependent [76]. We have largely shown in our studies on age-related diseases, such as CMD, how age can significantly affect the results obtained [77]. Age modulates any mechanism, activity, phenotype of cells [77], influencing for example the circulating lipidic [78] and protein profile, the levels of glucose, hormones, activity of microbiome and their metabolites, inflammatory and immune profile [79], and architecture and function of organs and systems [80]. Given its relevance it is imperative to consider this factor in selecting the population studied.

Concerning therapeutic treatments, it has been demonstrated by clinical trials with statins, β-blockers, and antihypertensive agents that individuals with poor persistence had worse outcomes than did persistent individuals [81,82]. Another study has evidenced that the activity of crucial pathways related to heart functionality are modulated by drugs. It has also been reported that the combined use of aspirin, a statin, and blood pressure lowering agents (polypill components) reduces the risk of vascular morbidity and mortality in patients with coronary artery disease [83].

These observations have motivated researchers to search for important recommendations to resolve this issue that could affect the search of accurate CMD biomarkers. For example, Howards [84,85] has recently recommended that an initial strategy might be design phase restriction, that consists of including a specific category of a confounder (e.g., male or female people); secondly, matching of controls to cases to enhance equal representation of subjects with certain confounders among study groups; then, performing analytical phase stratification, where the sample is divided into subgroups or strata on the basis of characteristics that are potentially confounding the analysis (e.g., age). To this point, it recommends performing a statistical adjustments regression for estimating the association of each independent variable with the dependent variable (the outcome) after adjusting for the effects of other variables. It can also consider the propensity score, which is a score given by the conditional probability of exposure to an intervention linked to a set of observed variables that may influence the likelihood of exposure. It also recommends including the instrumental variable, which represents a pseudo-randomization method. It divides patients according to levels of a covariate that is associated with the exposure but not associated with the outcome.

## 5. Another Revolutionary Approach to Consider in CMD Biomarkers Research: Extracellular Vesicles

**Figure 6:**
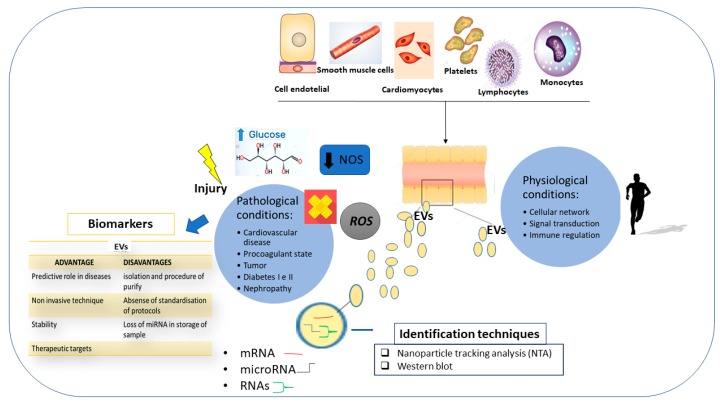
The roles of extracellular vesicle (EV) in physiological and pathological conditions; techniques for the identification of EVs; and advantages and disadvantages of their use in clinical practice.

Extracellular micro- and nanoscale membrane vesicles are produced by different cells and are attracting the attention of the scientific community. They act as mediators of intercellular communication and transport genetic material and signaling molecules to the cells. Accordingly, extracellular vesicle (EV) is comprised of not only membrane proteins and lipids from the cell surface, but also nucleic acids (DNA and RNA) including mRNAs, microRNAs, small-interfering RNAs, and long non-coding RNAs from the intracellular environment [86,87]. In the context of keeping homeostasis, the extracellular vesicles contribute to the regulation of various systemic and local processes. Significant advances have been made regarding the criteria of the following classification of the vesicles according to their origin, content, and function: Exosomes; microvesicles, also referred to as microparticles or ectosomes; and large vesicles defined as actively released vesicles [86,87]. Additionally, apoptotic bodies represented by a highly heterogeneous population of particles produced during apoptosis, the programmed cell death, also exist. However, a joined term “extracellular vesicles” (EVs) has been recommended for the definition of vesicles isolated from either the cell culture supernatants or the body fluids [86,87]. Because the content of EVs reflects the content of the cell of origin, multiple studies on EVs from body fluids in the context of disease diagnosis, prediction, and prognosis have been performed, actively supporting their high potential as a biomarker source. In the CMD studies, EVs related to thrombosis, inflammation, endothelial dysfunction, or angiogenesis, have been significantly observed in patients with acute coronary syndrome [88], ST-elevation myocardial infarction [89,90], cardiac remodeling [91], type 2 diabetic mellitus [92], diabetic retinopathy [93,94], as well as other CMD. They can be identified in body fluids, including blood, urine, synovial fluid, and many other body fluids, from individuals affected by various diseases. This suggests that they might represent ideal biomarkers for no invasive CMD diagnosis and prognosis. However, the true problem in this application is in the development of reliable and effective methodologies for routine laboratory analysis of EVs, as well as the discovery of the mechanism involved in the transport of genetic components from the cell environment into the EVs [95,96]. Consequently, it remains unclear and it is not easy to test EVs in liquid samples, or precisely in liquid biopsy. Furthermore, another crucial point in this analysis is in obtaining quantitative levels of genetic contents adapted to be tested, because the number of MVs is very low in the biofluids or they are loss during sample processing. Another matter is the eventual presence in the biofluids of food intake, medications, or other physiological and pathological factors. Thus, optimization of this assay is imperative, as well as biofluid collection, both of which require further research efforts (Figure 6).

## 6. Conclusions and Perspectives

In this review, we propose the integration of multi-omics approaches as a promising tool for the identification of appropriate biomarkers and therapeutic targets for CMD. This idea has been derived from the consideration that current omics studies on CMD have only focused on individual omics types. This represents a limitation in the deep understanding of molecules related to CMD mechanisms, because the separate omics evaluations enable the discovery of only a part of the CMD-related molecules, reducing the possibility of identifying well-fitted biomarkers. In addition, a significant number of past and current omics studies on CMD analyze the transcriptomic, epigenomic and proteomic profiles, that are currently considered routine. The analysis of metabolomics, microbiomic, and nutrigenomic profiles are only just now emerging, and are leading to the identification of some promising biomarkers (some examples have been mentioned above). Executing all the omics analyses in the CMD studies can certainly imply the detection of well-fitted biomarkers, obtaining data from multidimensional levels. This could also facilitate the complex management and therapy of CMD, the most important objective of clinical community. A further help in such research may also be derived from gender medicine that could add another crucial level in the differential CMD management and therapy according to gender. Today, gender represents one of the major CMD challenges. Having panels of appropriate biomarkers for CMD, and for gender, could also facilitate the design of appropriate algorithms for their prevention, diagnosis, and outcomes, as well as for the development of innovative guidelines. Certainly, such investigations require an appropriate design for eliminating confounding factors (e.g., age and medication, see paragraph 4), innovative models (i.e., 3D and 4D systems), and technologies (e.g., CRISPR/Cas9 and chemically modified RNA technique), as well as (given the complexity of multidimensional data) integration data tools, such as clustering and dimensionality reduction-based methodologies, predictive modeling approaches, pairwise integration, network-based methodologies, and composite approaches.

However, this field of research currently continues to be young, and consequently needs a major interest and more investigation, based on more accurate, standardized omics techniques. Nevertheless, the approaches and techniques suggested can give a detailed idea of the information about which gene, epigenetic factor, protein, pathway, metabolite, microbioma component, or environmental factor is helpful to consider in the investigation, as well as hypothetically detecting innovative biomarkers or targets. Cotemporally, this appraisal improves our appreciation versus the relevance of CMD pathophysiology in the research, given the rationale of how it may be translated into appropriate opportunities for clinical utility. Specifically, this investigation enhances our capacity to predict future CMD, categorize them on a molecular basis for a better diagnostic and prognostic management, and project personalized therapies tailored to the individual. However, as stressed, diverse questions remain to be addressed in order to benefit advances in personalized medicine, but well-designed investigations with the innovative techniques described could certainly support researchers to overcome them.

## Abbreviations

BCAA	branched-chain amino acids
CMD	cardiometabolic diseases
CRISPR/Cas9	clustered regularly interspaced short palindromic repeats -associated protein 9
GWAS	genome-wide association studies
MVs	microvesicles
NO	nitric oxide
PI3K/AKT/mTOR	phosphoinositide 3-kinase/protein kinase B/mammalian target of rapamycin
TLR	toll-like receptor
TMA	trimethylamine
TMAO	trimethylamine N-oxide
